# Machine learning workflow for edge computed arrhythmia detection in exploration class missions

**DOI:** 10.1038/s41526-024-00409-0

**Published:** 2024-06-22

**Authors:** Cyril Mani, Tanya S. Paul, Patrick M. Archambault, Alexandre Marois

**Affiliations:** 1 Faculty of Medicine and Health Sciences, McGill University, Montreal, QC Canada; 2 Thales Research and Technology (TRT) Canada, Québec, QC Canada; 3 Department of Family Medicine and Emergency Medicine, Université Laval, Québec, QC Canada; 4 School of Psychology, Université Laval, Québec, QC Canada; 5 School of Psychology and Humanities, University of Central Lancashire, Preston, Lancashire United Kingdom

**Keywords:** Electrodiagnosis, Preventive medicine, Medical research, Electrical and electronic engineering, Biomedical engineering

## Abstract

Deep-space missions require preventative care methods based on predictive models for identifying in-space pathologies. Deploying such models requires flexible edge computing, which Open Neural Network Exchange (ONNX) formats enable by optimizing inference directly on wearable edge devices. This work demonstrates an innovative approach to point-of-care machine learning model pipelines by combining this capacity with an advanced self-optimizing training scheme to classify periods of Normal Sinus Rhythm (NSR), Atrial Fibrillation (AFIB), and Atrial Flutter (AFL). 742 h of electrocardiogram (ECG) recordings were pre-processed into 30-second normalized samples where variable mode decomposition purged muscle artifacts and instrumentation noise. Seventeen heart rate variability and morphological ECG features were extracted by convoluting peak detection with Gaussian distributions and delineating QRS complexes using discrete wavelet transforms. The decision tree classifier’s features, parameters, and hyperparameters were self-optimized through stratified triple nested cross-validation ranked on F1-scoring against cardiologist labeling. The selected model achieved a macro F1-score of 0.899 with 0.993 for NSR, 0.938 for AFIB, and 0.767 for AFL. The most important features included median P-wave amplitudes, PRR20, and mean heart rates. The ONNX-translated pipeline took 9.2 s/sample. This combination of our self-optimizing scheme and deployment use case of ONNX demonstrated overall accurate operational tachycardia detection.

## Introduction

NOW fifty years after the last Apollo mission, NASA and its partners are planning the next exploration class efforts to send humans into deep space. The first objective is to create a sustainable human presence on the Moon and its orbit with the Lunar Gateway station. This outpost will serve as a stepping stone slightly outside of Earth’s gravity well for missions toward Mars and beyond^1^.

However, this new deep-space context breeds a new set of healthcare challenges to human explorers. Long-duration deep-space missions severely restrict ground resources capacity for medical assistance^2^. Previous low-Earth orbit methods to attend to the crew’s health, such as emergency or re-entry are no longer feasible^3^ in terms of spacecraft velocity change required when astronauts are in Trans-Lunar Injection and 450,000 km from Earth. Additionally, if an emergency arises during an orbit around the Moon with a capsule on its far side, the astronauts are completely isolated from communicating with the flight surgeons for periods up to 34 min^4^. In future Mars missions, the roundtrip communication delay of up to 44 min^5^ also disqualifies recent advances in live augmented reality telemedicine with the crew’s flight surgeon. In Mars missions, the closest emergency supplies and help are at best 7 months away^6^.

This healthcare delivery context is challenging since space and its microgravity environment are widely known for detrimental effects on humans’ health. Studies observe severe reductions in bone tissue through increased resorption, muscle mass atrophy linked to the lack of gravity, lung volume decrease through the cranial displacement of the diaphragm and abdomen, degradation of eyesight caused by the Spaceflight Associated Neuro-Ocular Syndrome and drop in red blood cell count through microgravity-caused hemolysis^7^.

Moreover, the literature reports that the spaceflight environment leads to cardiovascular system degradation in the absence of adapted countermeasures^8^. This degradation includes a decrease in left ventricular mass^9^, changes in atrial structure/electrophysiology^7,10^ and loss of overall cardiac contractility^7^, all of which predisposes the heart to arrhythmias. Microgravity conditions are also associated with cardiac functional alterations due to hemodynamic changes. These include fluid and pressure redistributions and a decrease in plasma volume, which may set the stage for changes in heart rhythm^9^, also potentially inducing arrythmias. For instance, the absence of gravity and associated hydrostatic gradients increases the mean arterial pressure in the torso while hypobaric and hypoxic environments considered for future exploration-class missions^11^ decrease the partial pressure of O_2_. Both effects can induce tachycardia as the heart attempts to redistribute pressure and supply sufficient oxygen^12^. Another potential arrhythmic pathophysiological pathway in space flight is through cosmic radiation exposure, which can cause inflammation and a decrease in microvascular density^13^. Cellular damage can then result from oxidative stress and tissue ischemia. The radicals produced by the oxidative processes accelerate the development of atherosclerosis and enhance plaque formation, particularly in the coronary ostia. Macrophages eliminate the injured cells, which are then replaced with fibrosis. This fibrosis and calcification can then alter the excitation conduction system and manifest clinically as cardiac arrhythmias^14^. Finally, ventricular arrhythmias during the Apollo program were linked to a reduction in total body potassium levels due to increased urinary excretion—a potential outcome of muscle atrophy during spaceflight^15^. Thus, overall, spaceflight and high-altitude environments could be arrhythmogenic through four key mechanisms of influence on the cardiovascular system: electrophysiological changes, structural alterations, hemodynamic changes, heart tissue composition changes, and electrolyte disturbance^9,15^.

This hypothesis aligns with numerous reports of observable cardiac arrhythmias in spaceflight environments^16^, ranking as the second most frequent medical issue during the Mir program era^15^ and even prompting an earlier return of a crewmember from Mir in 1987^17^. However, these recorded rhythm changes had no reported additional physiological adverse effects. No causal link between the cardiac pathology and the above mechanisms has also been empirically demonstrated^16^ since no deep-space missions past Earth’s magnetosphere have systematically studied these mechanisms of action through long-term electrocardiograms (ECG). Astronauts are also screened for perfect cardiovascular health^7^, making physiological challenges rarer to occur and notice. Thus, investigating and monitoring this gap in research can help understand and mitigate how new deep-space missions can aggravate cardiac fibrosis, contractility loss, atrial structure, and potentially affect heart rhythm variations. If arrhythmias are undetected and untreated, they could cause serious mission-threatening complications (i.e. strokes and heart failures)^18^.

Solutions must be found to increase space crew medical autonomy by empowering them with more on-board tools to monitor, study, detect and address treatable and degenerative cardiovascular conditions^19^. Focusing on early diagnosis and prevention instead of a reactive approach would also allow for earlier treatment of less severe conditions and increases chances of recovery and mission success^20^. Potential solutions exist through the introduction of wearables such as the Astroskin garment (Carré Technologies) and self-treatment software with integrated medical decision-support systems (MDSS)^19,21^. However, they could be more effective when combined with machine learning (ML) models. Thus, a solution resides in developing predictive models for in-space pathologies using biometric data streamed from such biomonitoring wearables.

Such an approach could enhance the awareness of the crew of pathologies developed on-board and enable faster reaction time in case of medical emergencies^19^. Here, early detection enables faster mitigation of symptoms and the start of treatment plans before ripple effect degradation^22^. This approach is recognized as a priority by governmental space agencies where, for example, a series of concept studies were issued by the Canadian Space Agency (CSA) as part of the Health Beyond Initiative where bio-diagnostics and decision-support systems were two of four technology development areas of interest (https://www.asc-csa.gc.ca/eng/funding-programs/programs/stdp/contracts-awarded-for-the-development-of-health-technologies-for-deep-space-missions.asp).

However, models must be deployed directly to the astronaut end-user in deep space, to allow more independence from ground resources^19^. In the context of Mars missions, the roundtrip communication delays pose a significant challenge in medical emergencies. Research by Romero and Francisco indicates that out of 100 medical problems likely to occur during deep space missions, about 31 to 42 require immediate treatment^23^, with 14 to 32 being acute, life-threatening emergencies like angina, anaphylaxis, or sudden cardiac arrest, necessitating response within 15 min^24^. This urgency underscores the need for the crew, particularly the Crew Medical Officer (CMO), to be autonomous in providing medical care, as they cannot rely on ground-based support. However, non-physician crew members often lack the training for complex medical diagnoses, placing substantial responsibility on the CMO. In cases where the CMO is incapacitated or unable to respond, it represents a critical single point of failure in the medical response pipeline. NASA’s Human Research Program (HRP) has identified this as a key scenario in medical system design, requiring non-physicians to administer emergency care^25^. One of such scenarios highlighted by NASA’s Exploration Medical Capability (ExMC) involves the CMO experiencing an irregular heartbeat during treadmill exercise, with a significant communication delay from Mars orbit. In such a situation, the crew must rely on an autonomous on-board diagnostic system, which needs to classify the irregular heartbeat in real time, immediately stop the treadmill to prevent further injuries and guide non-physician crew members through emergency responses, such as using an Automated External Defibrillator^26^. This scenario emphasizes the necessity for real-time, independent diagnostic capabilities on board the spacecraft’s limited computational resources, achievable through edge computing. While these on-board ML computers must be edge-optimized to be as compact and power efficient as possible to limit consumption of the scarce solar energy produced on deep-space vehicles (smaller solar arrays and weaker solar power in Mars), the case is even stronger during Extra Vehicular Activities (EVAs) or planetary surface explorations where connection might be lost to the main spacecraft systems (underground exploration, crater prospection, etc.) which would require to run these medical predictive models locally to the astronaut’s spacesuit, on portable smart devices.

Edge computing is a model design philosophy, which in this case, through tailored algorithms and efficient architectures, facilitates the processing and analysis of medical data at the points of care by enhancing ML model efficiency and enabling operation on various low-resource devices at the point of care like flight computers and smartphones. This increases ML model interoperability and deployability which has been a main focus of the CSA in the 2019 request for proposals (https://www.asc-csa.gc.ca/eng/funding-programs/programs/stdp/contracts-awarded-for-the-development-of-health-technologies-for-deep-space-missions.asp). In the context of limited on-board computational resources, this approach improves the resilience of the medical diagnosis pipeline while allowing for more accurate and quicker diagnoses and reducing reliance on Earth-based connectivity to make real-time decisions about patient care^19,27^on board the spacecraft edge computing servers and spacesuit integrated smart devices. Examples of edge architectures that enable such edge processing and analysis of data in real time include AWS Greengrass, Microsoft Azure IoT Edge, and Google Cloud IoT Edge^28^. These are made possible through containerization, which packages applications and their dependencies in a container, for more efficient deployment. ML frameworks allowing inference on edge devices include TensorFlow Lite (TFLite), OpenVINO, Caffe2 and ONNX Runtime^28^ (https://onnxruntime.ai/docs/).

Although various biometric models exist, not all exhibit such flexible and edge-compatible architecture that is crucial in the deep-space context. This limitation underscores the efforts of NASA’s ExMC, which is actively pursuing the development of real-time on-board ML medical diagnostic solutions such as the Exploration Medical Capability Clinical Decision Support System Concept of Operations (ExMC CDSS ConOps) which is specifically designed for deep-space missions and as required in its L4.1-Lunar-CDSS-0053 requirement^29^, integrates compatible edge-processing architectures for on-board processing capabilities.

However, in deep-space medical emergencies, it is essential to strike a balance between optimizing the efficiency of edge processes and maintaining the interpretability of the ML model’s inferences. Here, interpretability refers to both the model’s intrinsic ability to be understood (human-intuitive decision process, etc.) and the generation of supporting information (confidence intervals, feature importance, etc.) that can enhance the interpretability of the MDSS inference, though may induce additional computation time. On one hand, faster edge processing allows for near-real-time inference at the point of care on devices within the same vehicle as the patient, preventing further degradation of the patient’s condition in critical moments. On the other hand, the interpretability of the system ensures that the right decisions are made in situations where mistakes can be extremely costly. For example, with a transparent and interpretable model, the algorithm’s decision process can be easier to understand by a non-medically trained crewmember and the CMO can confirm that the diagnosed pathology is correct by quickly comparing this decision process to their own expert diagnosis. Thus, finding the right balance between both edge inference speed and interpretability can reduce overall medical execution time as they respectively lower reaction time on symptom appearance and increase trust in the MDSS pipeline, accelerating execution. In consequence, ML analysis interpretability and transparency are included as a NASA CDSS requirement which defines transparency as the generation of additional information that can enhance interpretability inference (L4.1-Lunar-CDSS-0050)^29^. Thus, interpretability remained a central criterion throughout this work, guiding design decisions due to its indispensable role in medical autonomy and decision efficiency.

For example, while deep learning (DL) has shown promising results in detecting tachycardia in ECG signals^30^, it often lacks this crucial transparency in its categorization processes, frequently functioning as a ‘black box’ despite recent advances in interpretability^31^. In our context, the goal was not to discover new relationships through black box algorithms, but rather to enable the inference of tachycardia from interpretable features whose relationship could be visualized and understood by the CMO for reasons outlined above. Thus, DL models were not included in this work and the CDSS currently being developed by NASA requires the use of ML (L4.1-Lunar-CDSS-0069), with no mention of DL requirements^29^. Additionally, DL’s inherent high computational requirements relative to ML make it less optimal for edge device deployment in a real-time inference perspective, reducing its relevance to the scope of this study. DL would increase crucial computation time in emergencies and rely on larger, dedicated computers sensitive to solar flares and cosmic rays, thereby introducing single-point failures in the medical pipeline compared to the ML edge computing alternatives.

In this work, tachycardias will be used as a case study to demonstrate such a deep-space bio-monitoring predictive approach. They are great candidate pathologies since they are detectable with lightweight ECGs integrated in an already long history of wearable cardiac biomonitors flown to the International Space Station (ISS) and Mir^16^. Tachycardias are also mostly treatable once detected with a range of accessible drug and cardioversion therapies^32^, but can seriously deteriorate if not acted upon^18^. Thus, this work could have a measurable positive impact on mission and health outcomes if implemented.

Specifically, this first stage to our research has focused on Atrial Fibrillation (AFIB) and Atrial Flutter (AFL). AFIB was selected as it is the most prevalent kind of tachycardia, characterized by erratic, chaotic electrical impulses in the upper chambers of the heart (atria), which result in a rapid heartbeat^33^. AFIB may be transient, but some episodes do not resolve without treatment. AFIB treatment protocols are feasible by medically trained astronauts as they can be simply addressed with a cocktail of rate control medication (metoprolol, diltiazem, or digoxin), rhythm control medications (procainamide, vernakalant, ibutilide, propafenone, or flecanide) and direct oral anticoagulants (dabigatran, rivaroxaban, apixaban, edoxaban) or warfarin^34^. AFL episodes, similarly to AFIB, may resolve on their own but can require treatment to avoid further symptoms. However, the ECG of patients with AFL is more organized than AFIB and maintains a regular heart rate^35^. AFL is treated similarly to AFIB. Both types of tachycardia are diagnosed using an ECG and have very similar signal processing, denoising requirements^36^ and demonstrate repeated recognizable patterns eligible for an ML approach.

This work reports the development and evaluation of the proposed biomonitoring predictive model approach using an ONNX-formatted machine-learning model pipeline approach. Our specific case study demonstrates the detection of periods of normal sinus rhythm (NSR), AFIB and AFL from a wearable 2-lead ECG. To define the needs of a ML pipeline optimized for such deployed deep-space inference, this paper will thus present the validated ML model algorithm and strategy highlighting six main contributions:

1. The demonstration of state-of-the-art denoising and preprocessing approaches for data extracted from physically active patients wearing bio-monitoring devices in a spacecraft’s high instrumentation and electromyographic noise environment while minimizing edge-computing requirements;
2. The implementation of ECG morphological and temporal feature extraction methods;
3. The development of a versatile triple-nested hyperparameter, parameter, and feature training scheme for a modular multivariable ML pipeline;
4. To our knowledge, the very first use of the ONNX format at the intersection of healthcare and deep-space edge computing;
5. The demonstration of ONNX-optimized inference performance on an Android edge device available in a deep-space context; and
6. The validation of this ONNX model performance against cardiologist labelling and the specific performance in detecting NSR, AFL and AFIB rhythms from raw data.

Additionally, our ML pipeline defined and ranked the highest impact features and hyperparameters to obtain high-accuracy NSR, AFL and AFIB identification.

## Methods

### Pipeline overview

**Fig. 1:**
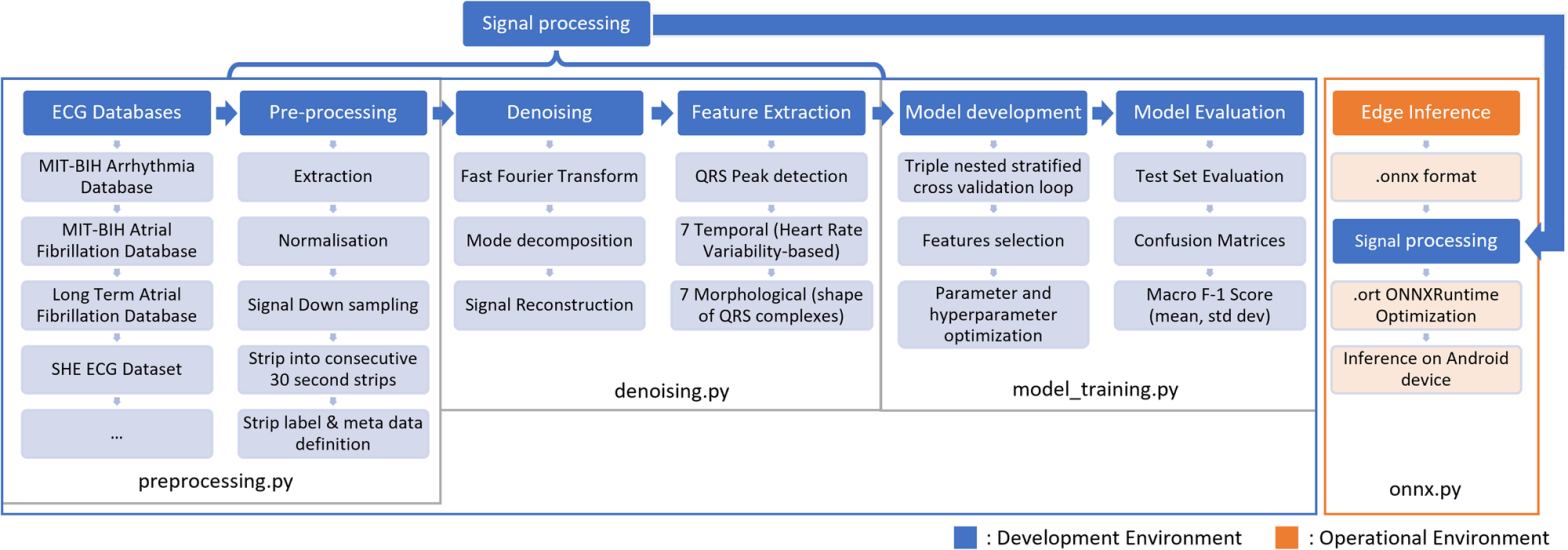
Overview of the machine learning development and operational pipeline. This figure illustrates the workflow used for ECG signal processing and machine learning, seperating the development (blue) to the operational (orange) environment flows. In the development environment, the workflow starts with ECG data sourced from various databases, followed by pre-processing steps like extraction, normalization, and down-sampling. The denoising stage applies techniques such as Fast Fourier Transform and signal reconstruction. Feature extraction focuses on detecting QRS peaks and deriving temporal and morphological features. Model development involves cross-validation, feature selection, and hyperparameter optimization, leading to model evaluation through test set evaluation and confusion matrices. In the deployed operational flow, the trained model is converted to the ONNX format, optimized with ONNX Runtime for edge inference and then uses the signal processing workflow of the development environemnt to feed operational data to the model. This allows results in the target edge inference on an Android device. Arrows indicate the sequential progression from data acquisition through processing, model development, and final deployment which is managed through Python scripts, ensuring a seamless transition from development to deployment.

As shown in Fig. 1, the pipeline used in the development and inference environments of the ML model is divided into seven main components: Databases, Pre-processing, Denoising, Feature Extraction, Model Development, Evaluation and Edge Inference. This pipeline was designed as a modular system, allowing for additional databases, features and continuous improvements to the process independently of the rest of the system. Figure1 will be referred to in the next sections, which will dive into the methods used in each step of the signal processing pipeline.

### Databases

In the signal processing pipeline development, two types of databases were used. The first provided a range of ECG recordings with various levels of natural and artificial noise to fine tune the denoising process for an analogue aerospace noise environment. The second set focused on collecting NSR, AFIB and AFL rhythms to train the model.

#### Denoising databases

This first subset of datasets is a collection of ECG recordings of which the leads were separated. These recordings were selected for their noise features; they are all from monitored patients in a state of exercise, stress and surrounded by electrical equipment, which created analogue noise sources similar to a deep-space monitoring context. All these datasets also contain ECG recordings of patients at rest thus providing a reference for the denoising process covered in a later section.

1. MIT-BIH Noise Stress Test Database (https://physionet.org/content/nstdb/1.0.0/)^37^: Twelve half-hour ECG recordings and three half-hour noise recordings that are characteristic of ambulatory ECG recordings were recorded.
2. MIT-BIH ST Change Database (https://physionet.org/content/stdb/1.0.0/): 28 ECG recordings of varied durations, the majority of which were made during exercise stress tests and show transitory ST depression, are included in this database.
3. EPHNOGRAM (https://physionet.org/content/ephnogram/1.0.0/): An electrocardiogram and phonocardiogram database created from 24 patients aged 23 to 29 years old who participated in 30-minute stress tests with fitness equipment.

#### Arrhythmia databases

The arrhythmia datasets were used for the model training phase. They are all supervised, meaning they have rhythm annotations independently made by cardiologists following the Association for the Advancement of Medical Instruments convention^38^, indicating each heartbeat superclass and each rhythm segment behavior for NSR, AFL and AFIB among others. A large majority of these datasets originate from the Arrhythmia Laboratory at the Beth Israel Deaconess Medical Center, Massachusetts Institute of Technology. They have been obtained through the open-source PhysioBank repository, a digital archive maintained by the United States National Institutes of Health. The MIT Databases have shown to be performant in training a ML model^39^.

1. MIT-BIH Arrhythmia Database (https://physionet.org/content/mitdb/1.0.0/)^40^ (MITDB): 48 half-hour ambulatory ECG recording snippets from 47 participants are included in this dataset. A total of 4,000 24-hour ambulatory ECG recordings were taken from a population that included a mix of in and outpatients at a 360-Hz sampling rate.
2. MIT-BIH Atrial Fibrillation Database (https://physionet.org/content/afdb/1.0.0/)^41^: 25 long-term ECG recordings of people with AFIB are sampled at 250 Hz in this database (mostly paroxysmal).
3. MIT-BIH Normal Sinus Rhythm Database (https://www.physionet.org/content/nsrdb/1.0.0/): This database contains 18 long-term ECG recordings sampled at 128 Hz from individuals with no major arrhythmias; the patients include 5 men and 13 women between the ages of 20 and 50.
4. Long Term Atrial Fibrillation Database (https://physionet.org/content/ltafdb/1.0.0/)^42^: 84 long-term ECG recordings of people with paroxysmal or persistent AFIB are included in this database. Records can last anywhere from 24 to 25 h and are sampled at 128 Hz.
5. Chapman University and Shaoxing People’ Hospital Database (https://www.kaggle.com/datasets/bjoernjostein/shaoxing-and-ningbo-first-hospital-database): The dataset contains 45,152 labelled 10-second 12-lead ECGs at 500 Hz.
6. China Physiological Challenge in 2018 (https://www.kaggle.com/datasets/bjoernjostein/china-physiological-signal-challenge-in-2018)^43^: This dataset, gathered from 80 healthy individuals, contains continuous ECG recordings made over 24 h, totaling more than 200,000 annotated ECG at a 500-Hz sample rate.

Since model performance is highly dependent on the composition of data used, Table 1 reports the database-specific number of 30-second ECG samples for each studied cardiac rhythm, a subset of the original databases.

**Table 1:** Number of 30-second ECG samples for each cardiac rhythm label studied

Dataset	NSR	AFIB	AFL
MIT-BIH Arrhythmia Database	602	102	22
MIT-BIH Atrial Fibrillation Database	1275	1337	343
MIT-BIH Normal Sinus Rhythm Database	51,840	0	0
Long Term Atrial Fibrillation Database	22,834	7358	0
Chapman University and Shaoxing People’ Hospital Database	608	593	148
China Physiological Challenge in 2018	918	1098	0
Total	78,083	10,488	513

### Pre-processing

In the pre-processing step decomposed in Fig. 1, data is extracted from the different heterogenous databases with the *wfdb* library (https://www.physionet.org/files/wfdb/10.7.0/wpg.pdf) and is harmonized to a single normalized dataset that can be used in the next pipeline sections. First, the data is imported for all raw leads, patients and databases into a hierarchical data file format, here HDF5, using the *h5py* Python library (https://docs.h5py.org/en/stable/). This allowed for variable format datasets and variable-length strings—optimal for preprocessing of unequal length samples—by mapping contents to Python object arrays. Leads are separated, but the patient identification numbers are conserved as metadata for database properties used in ML functions (discussed in the Model Development and Evaluation section). The data was then cut into non-overlapping 30-second samples^44^. At this point, the HDF5 hierarchy integrated this metadata, turning into a parameter-driven ECG-sample database, which also tracked which samples are temporally consecutive to separate them downstream.

The ECG samples were then normalized first using the *wfdb* library to downsample at a 128-Hz standard rate to match the lowest database sample frequency of 128 Hz (LTAFDB)^42^. This standard frequency still respected Nyquist frequency requirements to avoid aliasing^45^ on important frequency ranges discussed in the next section. Invalid values were also removed by comparison with a rolling average, units uniformed thanks to a dictionary of standards, values centered on the x-axis by subtracting the mean and then scaled between upper and lower bounds set at 1 and −1. These last two steps allowed to pad shorter ECG samples with zeroes to maintain the standard 30-second length. Each ECG sample was then attributed a single-label rhythm definition as meta-attribute (AFL, AFIB, NSR) based on the original datasets’ cardiologist ECG rhythm annotations, thus enabling the supervision of the model in later steps. In case of conflicts, if the sample contained both an NSR and tachycardia label, the tachycardia label was maintained and if there are two tachycardia labels, the sample is discarded. Samples containing any other types of tachycardia than the ones studied were automatically discarded. The result of this step was a pre-processed synthesized database containing only relevant 30-second samples of the rhythms studied, which tracked the original database, original ECG number (patient), temporal order, lead, data, and arrhythmia label.

### Denoising and feature extraction

**Fig. 2:**
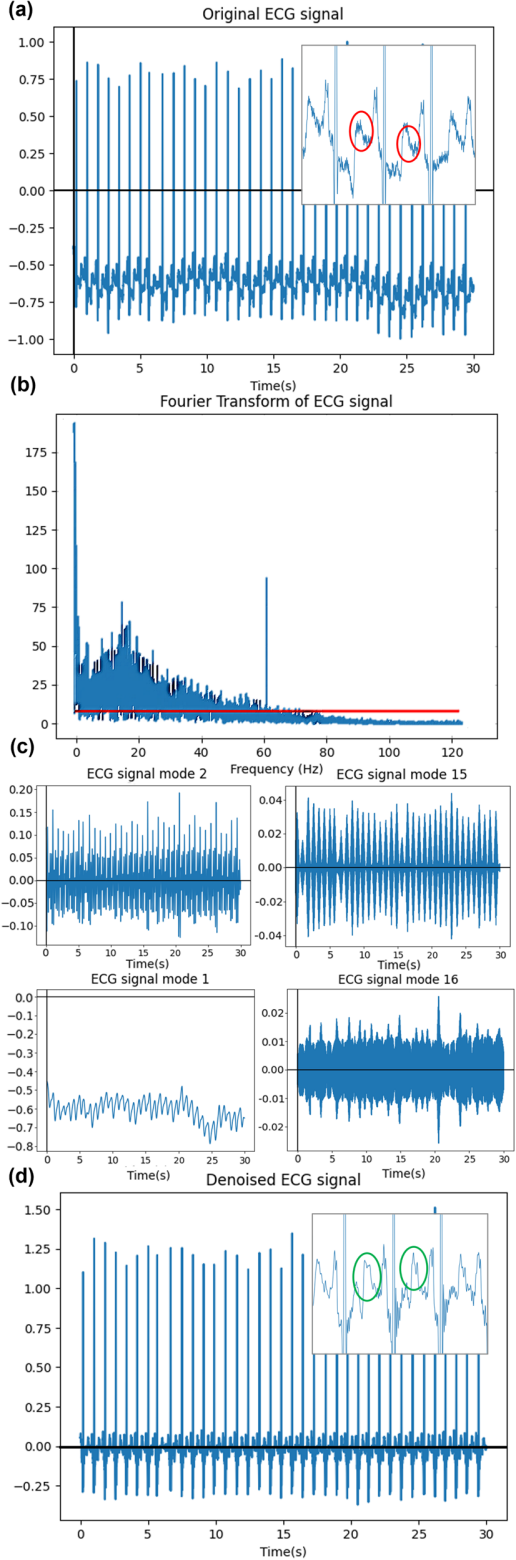
Step-by-step breadown of the custom denoising process. Visual illustration of the denoising process: **a** Original noisy ECG signal with noise artifacts circled in red. **b** Fast Fourier Transform of the noisy ECG signal, with mean spectral amplitude in red. **c** Top: Frequencies preserved on each edge of the reconstructed mode band. Bottom: Previous and subsequent modes, showing noise removal. **d** Denoised signal with cleaned features circled in green.

Considering the high electromagnetic noise interference environment on a spacecraft^46^, and the physical activity context in which the ECG data might be recorded, a foundation of this work was to develop an effective denoising procedure tailored to the deep-space edge computing context. To enable proper inference, this denoising algorithm detected and removed noise without compromising important biometric data, as shown in Fig. 2.

The first step of the denoising algorithm was to identify the noise to discard. Using the lunar gateway as a reference for new-generation deep-space spacecraft environments, its 400-Hz power supply will be converted to 60 Hz for experiments and commercial equipment^47^. Thus, three types of ECG frequencies need to be removed^48^: under 3–4 Hz (baseline wander noise, muscle artifacts, breathing), over 50–60 Hz (spacecraft power interface, instrumentation noise), and electrode motion artifacts. This is in line with literature raising the importance of maintaining frequency modes under 15 Hz that contain most ECG components (P and T waves) and modes between 30–60 Hz that contain most QRS complex information^36^. Thus, the frequencies between 4–60 Hz were conserved as shown in Fig. 2.

To reduce computational strain on space-based edge devices, the denoising algorithm started by detecting noise using a Fast Fourier Transform (with the *SciPy* library) to find the significant presence of frequencies under 4 Hz and above 60 Hz. The significance is defined by a larger magnitude than the mean of the border frequencies.

If significant noise was detected, the algorithm used Variational Frequency Mode decomposition (VFMD) with the *vmdpy* library, for signal deconstruction and reconstruction. In the literature, VFMD is the preferred choice for AFIB and AFL over other denoising algorithms like filtering or independent component analysis. It outperforms in post-cleaning QRS complex recognition, signal-to-noise ratio improvement^36,49^ and spectrum tailoring, a vital feature for handling yet undefined deep-space noise profiles.

Thus, with a resampling frequency of 256 Hz, the mode decomposition was evenly distributed between 0 and 128 Hz (Nyquist frequency^45^) with 32 modes (*K* = 32) of range 4 Hz each. The signal was then reconstructed using sub-bands 2–15 (4–60 Hz, *j* = [2, 15]).

Once the ECG signal denoised, 17 features were extracted (summarized in Table 2) which can be split into two categories: heart rate variability (HRV) features to track the time-domain intervals between the QRS complexes (min/max/mean heart rate, rMSSD, PRR50, PRR20, and SDSD, SDRR) and morphological features (P/Q/R/S/T median peak amplitude and P-Q/Q-R/R-S/S-T median peak delay) to track the shape of the QRS complexes. These are conventionally used metrics in cardiology, chosen to enable tachycardia inference from features whose impact and relationships would be familiar to a CMO with basic cardiology training, as opposed to model-generated abstract signal behavior features^50^.

**Table 2:** Summary Of HRV And Morphological Features Used In The ML Model Training

Feature	Domain	Description	Equation	Unit
min, max, mean heart rate	Time	Heart rate maxima based on the time between all successive heartbeats measured from R-R peak intervals of denoised data.	$1 \, \text{beat} \times \frac{60 \left( \text{s}\, \text{min}^{-1} \right)}{\text{RR}_{\text{i}}(\text{s})}$	Beats per minute (BPM)
rMSSD	Time	Root Mean Square of Successive Differences between each R-R peak.	$\sqrt{{\rm{mean}}{[\left({{\rm{RR}}}_{1}-{{\rm{RR}}}_{2}\right)}^{2}+\ldots +{\left({{\rm{RR}}}_{{\rm{i}}}-{{\rm{RR}}}_{{\rm{i}}+1}\right)}^{2}]}$	milliseconds
PRR50	Time	The proportion of consecutive R-R intervals that differ by more than 50 ms.	$\frac{n\,{\rm{of}}\left|{{\rm{RR}}}_{{\rm{i}}}-{{\rm{RR}}}_{{\rm{i}}+1}\right| > 50}{n-1}$	N/A (unitless)
PRR20	Time	The proportion of consecutive R-R intervals that differ by more than 20 ms.	$\frac{n\,{\rm{of}}\left|{{\rm{RR}}}_{{\rm{i}}}-{{\rm{RR}}}_{{\rm{i}}+1}\right| > 20}{n-1}$	N/A (unitless)
SDSD	Time	Standard deviation of the differences between successive R-R intervals.	${\rm{std}}[({{\rm{RR}}}_{1}-{{\rm{RR}}}_{2}),\ldots ,({{\rm{RR}}}_{{\rm{i}}}-{{\rm{RR}}}_{{\rm{i}}+1})]$	milliseconds
SDRR	Time	Standard deviation of all R-R intervals.	${\rm{std}}[({{\rm{RR}}}_{1},{{\rm{RR}}}_{2},\ldots ,{{\rm{RR}}}_{{\rm{i}}})]$	milliseconds
P, Q, R, S and T median peak amplitude	Amplitude	Respective amplitude of each wave of the median QRS peak.	${\rm{medXPeak}}=({\rm{Xpeaks}}[{\rm{medPeakIndice}}])$ X represents a P, Q, R, S or T wave	N/A (normalized)
P-Q, Q-R, R-S and S-T median peak delay	Time	Respective delay between each wave of the median QRS peak of the sample.	${\rm{XYdelay}}=\frac{\left({\rm{medXPeak}}-{\rm{medYPeak}}\right)}{{\rm{signal\; frequency}}}$ X, Y represent consecutive waves (P, Q, R, S or T)	milliseconds

### Model development and evaluation

#### Model type selection

The initial phase of model development involved choosing a model type suitable for deep-space arrhythmia inference. While initial experimentation was done with a multinomial regression model, a decision tree classifier was ultimately selected (demonstrating the pipeline’s modularity, as the architecture change took less than an hour). This complies with NASA’s L4.1-Lunar-CDSS-0060 requirement for deep-space clinical decision support system, which recommends logistic regression, decision tree and random forest amongt other ML algorithms^29^. Compared to the other suggested model architectures, this decision was driven by the fact that decision tree classifiers provide a more interpretable inference process for the CMO and the ability to assess feature importance while maintaining high accuracy—essential attributes in operational medicine^51^. In contrast, logistic regression tends to be less accurate^52,53^ and random forest, although accurate, is less interpretable^52^.

#### Training Procedure

At this stage of the pipeline, the HDF5 dataset contained a large set of normalized and denoised ECG samples each with 17 features and a rhythm label. These labels and associated features served as benchmarks during the training phases, and as validation statistics during the testing phases of the designed cross-validation approach in the supervised training procedure.

**Fig. 3:**
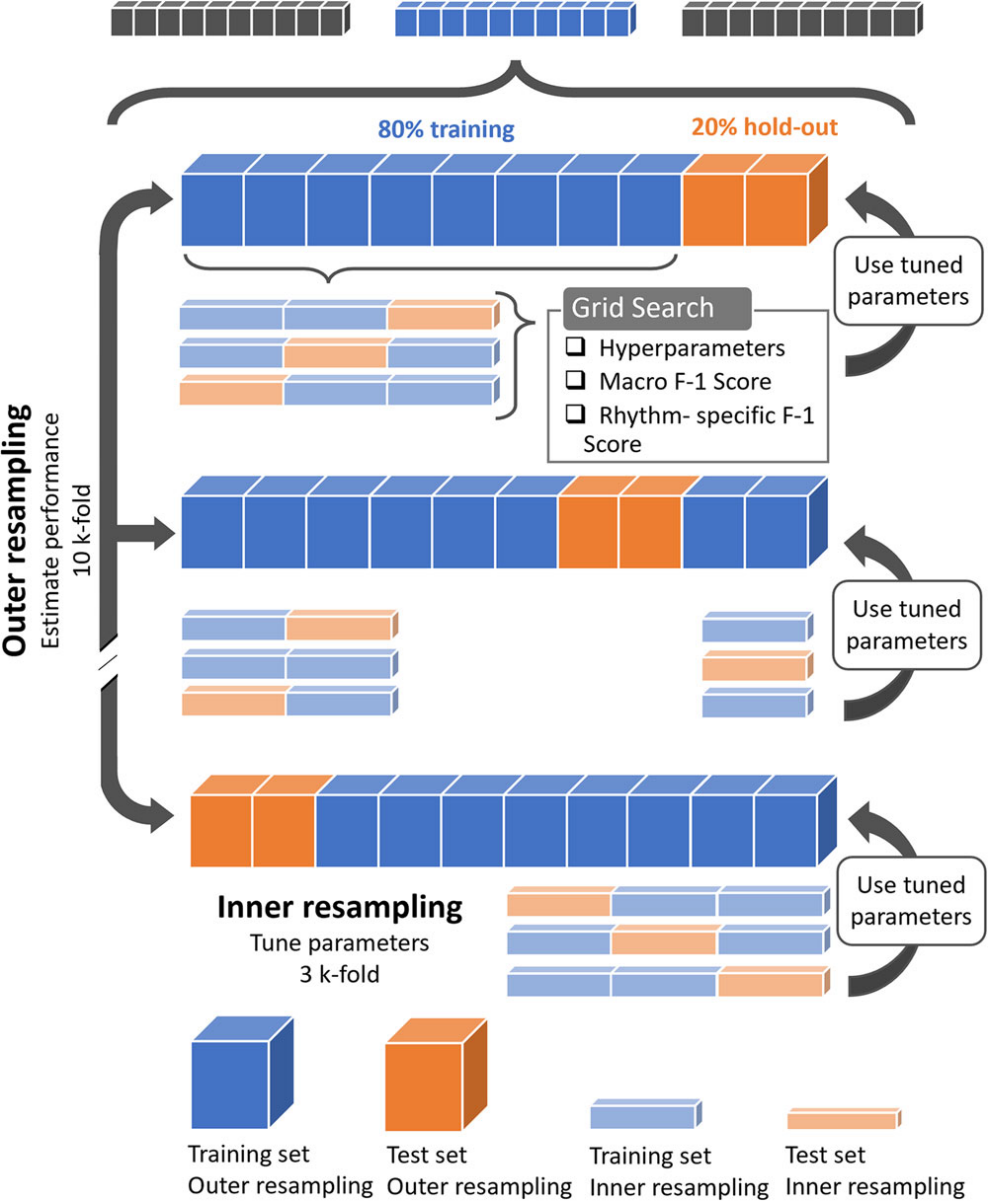
Flowchart of the model training and optimization process for parameters and hyperparameters. This figure illustrates the flowchart of the model training and optimization process for parameters and hyperparameters. The process starts with γ models, represented by the blue and grey sets at the top, each with different feature spaces. These models are trained and compared to identify the best-performing one. The main workflow involves an outer resampling process (10 *k*-fold cross-validation) to estimate performance. In this stage, 80% of the data (blue) is used for training, and 20% (orange) is held out for testing. Within the outer resampling loop, an inner resampling process (3 *k*-fold cross-validation) is used to tune parameters. Grid search is employed to optimize hyperparameters, macro F-1 scores, and rhythm-specific F-1 scores. The tuned parameters from the inner resampling are then applied to the training data in the outer resampling loop. Arrows indicate the flow of data from training to testing and the application of tuned parameters at each stage.

Cross-validation is a recognized approach to optimize model performance and avoid biases such as overfitting. There are different potential methods in cross-validation, such as simple *k*-fold, leave one out, stratified cross-validation or double cross-validation. Within the presented pipeline, we introduced nested subject-independent cross-validation specifically to reduce the bias in combined hyperparameter tuning and model selection by limiting exposure to a subset of the dataset provided by the outer cross-validation procedure^54^. This approach, combined with a holdout test data set for final model evaluation, simultaneously optimized for the features, hyperparameters and parameters of multiple model instances by comparing different configurations. The designed process consisted of six steps as seen in Fig. 3. 1) Prompting the user with the number of models (*γ*) to train and compare in a run as well as how many features (*η*) to track. To reduce model complexity, a subset of *η* features was randomly selected for each model out of the 17 available, creating *γ* feature description arrays and *γ* feature value arrays. A solution array was also created with the cardiac rhythm of each sample. This allowed us to extract label statistics for each run. The next steps were then performed for each *γ* model instances. 2) Stratified splitting of outer train and holdout test datasets at an 80%-20% ratio. 3) Starting an outer cross-validation on the training subset with *stratifiedKfold* of the *scikitlearn* Python library (https://scikit-learn.org/stable/). Here the dataset was split into equally distributed 10 folds and cross-validated for parameter tuning. 4) Nesting an inner stratified 3-fold cross-validation, where a grid search evaluated the model fit of different hyperparameters on the last fold based on a macro-F1 score. *RandomCV* of the *scikit* library was originally implemented to coarsely converge towards to a sensible hyperparameter domain after which *GridSearchCV* was deployed to result into the best model through finer hyperparameter tuning. 5) Returning the best hyperparameter fit to the outer loop to continue parameters tuning. 6) Finally, each *γ* model’s best fit (parameters and hyperparameters) underwent a last evaluation on the holdout set. The most performant model was then selected, and its characteristics (tracked features, parameters) recorded. The importance (*β*) of these features was also recorded, with the *feature_importances* function in *scikitlearn*.

Five methodologies were maintained across dataset operations to optimize training performance:

1. The seeds used in each part of this training procedure were stored in a separate file to ensure replicability.
2. To avoid over-fitting for patients or specific recordings, all samples from the same patient and recording were within the same subdivided training set.
3. Similarly, no temporally dependent samples were consecutive in the training sets.
4. At least one completely new ECG type (new patient and ECG recording source), on which the model was not trained, was held in the test dataset to validate how the model reacted to new ECG variations.
5. While the cross-validation stratification ensures that the properties between training sets were maintained, there were no forced proportionality on the ECG rhythms in the datasets. The model is trained on the same pathological proportions as it would be exposed to in a medical context to avoid overfitting for more rare rhythms (i.e., AFL).

The new randomization seed of each prompted splitting and reshuffling process was stored in the seed bank. Overall, this training procedure was designed to be modular, feature-agnostic, arrhythmia-agnostic and model architecture agnostic to avoid impeding future expansions to this work or on the interoperability feature of a downstream ONNX integration.

#### Model evaluation

The models were evaluated based on the confusion matrices generated by aggregating the final 10-fold cross-validation of the training set and by testing on the holdout set. From these matrices and their true positives (TP), false negatives (FN) and false positives (FP), the rhythm-specific Accuracy, Recall, Precision and F1 score were computed, where each rhythm was considered the positive class and the others negative.

For each model and their rhythms, the mean and standard deviation of this F1 score was obtained for the aggregated 10-fold cross validation and test set. The macro-F1 score was then calculated by obtaining the average of the mean F1 score for each rhythm’s cross validation and test set. The final selected model had to first pass the threshold F1 score of 0.75 for each rhythm—to ensure holistic performance—and was then selected to maximize the macro-F1 score. This method was chosen since F1 scoring was determined in literature as a more meaningful evaluation for imbalanced datasets, which ours is considering the low proportion of AFL in Table 1. The Results section contained a tabulated representation of the generated results on which the models were evaluated.

Additionally, since our triple nested optimization approach was designed to outperform regular ML pipelines, each decision tree model’s performance was compared to a baseline logistic regression pipeline throughout development. Logistic regression was selected as baseline as it is closest to decision trees in terms of interpretability, a central criterion^52^. This comparison was achieved by generating for both the development and baseline models a multiclass, one-vs.-all receiver operating characteristic (ROC) curve for each cardiac rhythm, where each rhythm was consecutively treated as positive and all the other classes as negative. These are then micro-averaged into one ROC curve to give a general sense of each model’s performance. Micro-averaging was used over macro-averaging as it is preferable for highly imbalanced dataset as it was the case with ours^55^. This imbalance is why weighted confusion matrices were used for evaluating the model since the NSR over-representation would obscure any relevant results in unweighted confusion matrices.

#### Selected model architecture

Following the optimization scheme presented in Fig. 3, the selected version of the decision tree classifier used the entropy criterion based on the Shannon information gain and the following hyperparameters: a maximum depth of 9, a minimum of 30 samples to split an internal node, a minimum of 5 samples to be at a leaf node and no class weight balancing per the non-proportional training rules to avoid overfitting.

### Edge inference

After the best scikit-kit learn decision tree model was selected, it was first converted to an ONNX format. ONNX is an open-source standard model format that can seamlessly import almost any popular DL and ML framework^56^ by supporting a wide variety of neural network architectures (https://github.com/onnx/onnx) without the need for extensive conversion, unlike TFLite. The philosophy of the ONNX approach is also to be platform-agnostic, which means that models trained on one platform can be deployed on a variety of edge devices all running different operating systems and architectures, as opposed to OpenVINO, which is optimized for Intel architectures. As mentioned in the Introduction, this interoperability of platforms and frameworks has been a priority for state space agencies, which wish to integrate multiple healthcare models from different development streams in central medical decision support platforms while avoiding compatibility issues or the need for separate computing devices and operating systems^19^. In that objective, the ONNX format is an essential tool to maximize interoperability, deployability and software architecture redundancy crucial to the edge inference context of restricted deep-space computational resources since any model can operate on any available device, hence being chosen as the format for this model.

The inference at the end user—here astronauts—is possible through ONNX Runtime, a lightweight open-source edge inference engine for ONNX format models. It first optimized the selected model for deployment on low-resource edge devices using a variety of techniques deployed through ONNX Runtime, including constant folding and node fusion/elimination to reduce memory and computation requirements (https://github.com/microsoft/onnxruntime). Then, it enabled the deployment of the model and operator configuration files on the device through mobile-configured containerization.

Overall, ONNX enables to bridge the gap between model development and deployment on edge devices without any loss of accuracy or performance, by enabling lightweight edge computing capacity for medical ML applications in deep space where power for computational resources is limited.

Considering that many flight-proven wearable bio-monitors use Android OS, this study demonstrated inference of the ONNX runtime model on an Android edge device as proof of the operational use case. This was achieved by first exporting the sklearn model to the ONNX format using the *onnxmltools* library (https://github.com/onnx/onnxmltools) and the *convert_from_sklearn* method. The ONNX model was then optimized as discussed above and translated into a dual-optimized model file (.ort) and operator configuration file (*onnxruntime* package). Note that while the Scikit or ONNX format do not inherently perform quantization or pruning, which was neither performed manually, it could be executed by ONNX Runtime as part of model compression algorithms. Both files were then deployed to the Android device. To do so, the Android Studio development environment was installed in Samsung S10 running Android 13. The ONNX Runtime library was then imported into the Android project as a module where it was included in the *build.gradle* file with all the necessary dependencies. Then, the ONNX runtime engine was initialized in the Android app, the ONNX model loaded and the input data provided for inference. The model was run with the ONNX Runtime engine, which processes the pre-recorded ECG input data and provided the inference service.

## Results

### Model development

**Fig. 4:**
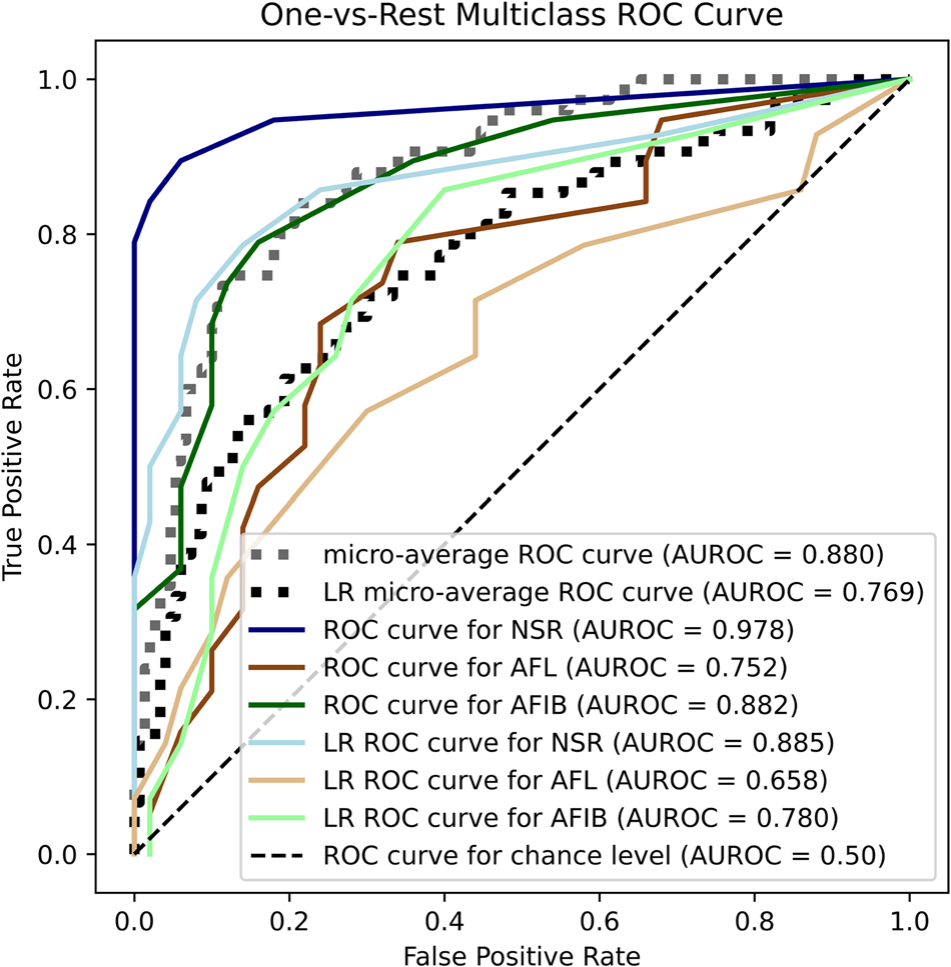
Receiver operating characteristic (ROC) curves of the developed model and a linear regression model to compare inference performance for each cardiac rythm studied. This figure is a visual representation of the inference performance of our model (depicted in dark colors) and a linear regression model (LR, depicted in lighter versions of each color) through a receiver operating characteristic (ROC) curve for the three cardiac rhythms studied. The plot includes ROC curves for Normal Sinus Rhythm (NSR), Atrial Flutter (AFL), and Atrial Fibrillation (AFIB), along with their respective area under the ROC curve (AUROC) scores. The ROC curve for the chance level is shown with a dashed line. The model’s micro-average ROC curve has an AUROC of 0.880, while the LR micro-average ROC curve has an AUROC of 0.769. Specific AUROC scores for each rhythm include 0.978 for NSR (model) and 0.885 (LR), 0.752 for AFL (model) and 0.658 (LR), and 0.882 for AFIB (model) and 0.780 (LR). The plot effectively demonstrates the superior performance of our model compared to the linear regression model across different cardiac rhythms.

Figure 4 compares the inference performance of the final version of the developed model and the classical logistic regression pipeline ran in parallel. Our model justified its added complexity by presenting a better area under the ROC curve (AUROC) in each category (NSR: 0.978 vs. 0.885, AFIB: 0.882 vs. 0.780 and AFL: 0.752 vs. 0.658, for our decision tree model compared to the logistic regression, respectively). Overall, the micro-averaged ROC curve for our model had an AUROC of 0.880 compared to 0.769 for the logistic regression.

### Model Inference Evaluation

**Fig. 5:**
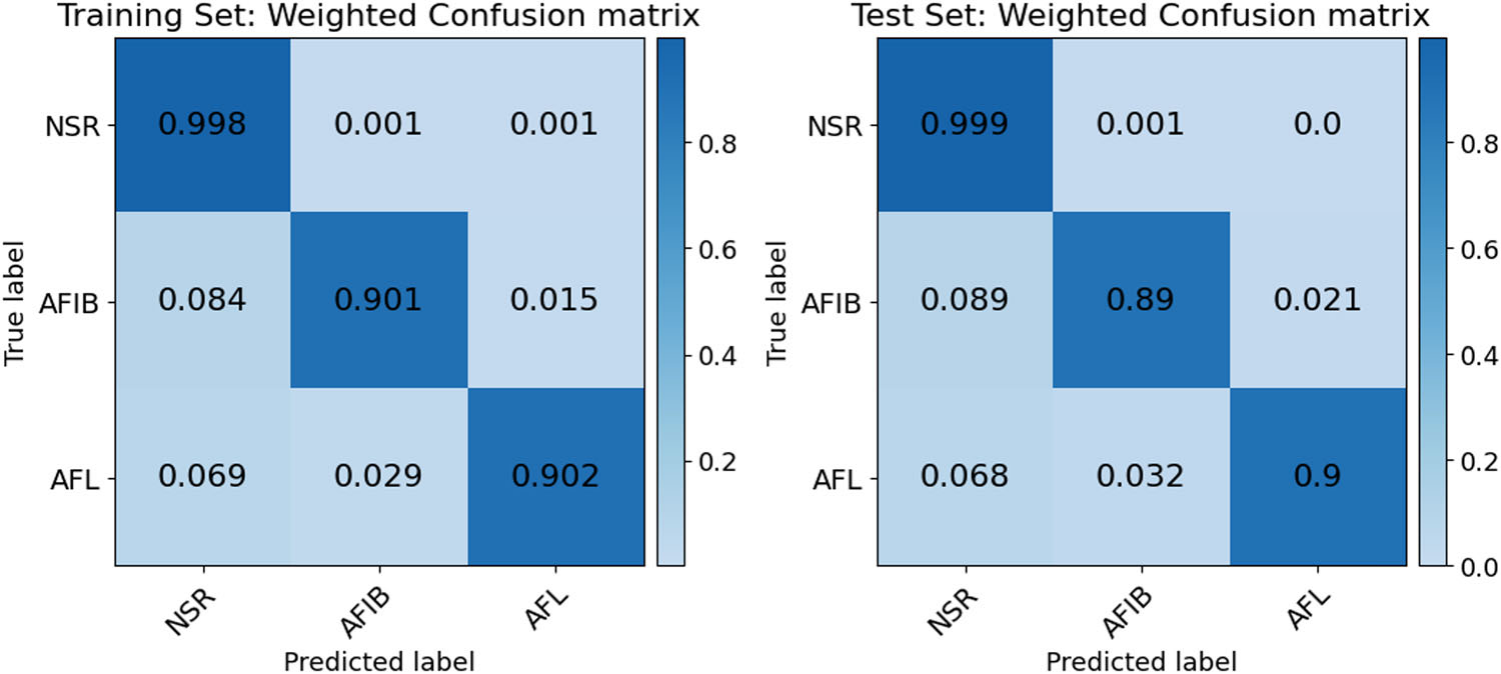
Weighted confusion matrices of the train sets (left), and holdout test set (right). This figure presents weighted confusion matrices for the training set (left) and the holdout test set (right). The confusion matrices display the classification performance of the model across the three cardiac rhythms studied : Normal Sinus Rhythm (NSR), Atrial Fibrillation (AFIB), and Atrial Flutter (AFL). Each cell in the matrices shows the proportion of true labels (rows) correctly or incorrectly predicted as specific labels (columns). High values along the diagonal indicate correct classifications, while off-diagonal values indicate misclassifications. For the training set, the model shows high accuracy with NSR (0.998), AFIB (0.901), and AFL (0.902) correctly classified. Similar performance is observed in the test set, with NSR (0.999), AFIB (0.89), and AFL (0.9) correctly classified. The color intensity reflects the classification accuracy, with darker colors representing higher accuracy.

Our model performance is further illustrated with Fig. 5. where weighted, multiclass cardiac rhythm confusion matrices show strong relative True Positive proportions in both the training set (NSR: 0.998, AFIB: 0.901, AFL: 0.902) and in the test set (NSR: 0.999, AFIB: 0.890, AFL: 0.900) as illustrated by the predominantly diagonal heatmap. This performance is also tabulated in Table 3 through F1 scoring of the confusion matrices. The selected model had a macro F1 score of 0.899 with specific cross-validation mean scores of 0.993 for NSR, 0.938 for AFIB, and 0.767 for AFL. The inference performance for each rhythm was thus above the 0.75 F1 score threshold. Following the training, the test set performance exceeds the 10-fold cross-validation mean with increases of F1 scoring for each rhythm as NSR detection scored 0.994, AFIB 0.944, and AFL 0.803. The main features the selected model tracked (and their associated feature importance *β*) were the P-wave median peak amplitude (0.69), PRR20 (0.14), mean heart rate (0.10), max heart rate (0.02), SDRR (0.02), PRR50 (0.02) and SDSD (0.01). Accuracy was high at 98.9% for NSR, 98.6% for AFIB, and 99.7% for AFL.

**Table 3:** Classification performance of the selected model

Cardiac Rhythm	Mean F1 Score (10-Fold CV)	F1 Score standard deviation (10-Fold CV)	Test Set	Mean Accuracy (%)
NSR	0.993	0.03	0.994	98.9
AFIB	0.938	0.03	0.944	98.6
AFL	0.767	0.11	0.803	99.7
Macro F1 Score	0.899	0.06	0.914	–

Additionally, the ONNX-adapted ECG pipeline only took 9.2 seconds per 30-second sample of raw data to run on the Android device. Thus, this allows sufficient time for the ECG recording to finish and the edge device to be ready for the next round of pre-processing and inference.

## Discussion

The objective of this work was to present the performance and detailed structure of an ML pipeline and model optimized for the detection of NSR, AFIB, and AFL rhythms in ECG recordings with the use of the ONNX format at the intersection of healthcare and deep-space edge computing. Overall, the modular nested training scheme and its resulting model based on morphological and HRV features provided accurate detection of tachycardia with lower identification of AFL compared to AFIB and NSR in raw ECG recordings.

The overall evaluation of the model shows a well-rounded performance with accuracies over 95%, a 14% higher AUROC micro average score compared to a logistical regression trained on the same dataset and a 0.914 macro-F1 score. It is important to compare these two last metrics, since optimizing the F1 score (i.e., the harmonic mean of precision and recall) allows to identify as many positive examples as possible, regardless of the number of false positives. On the other hand, AUROC is useful in the context of classification problems where the cost of false positives and false negatives is different^57^. In our case, both the AUROC and F1 scoring are high, and the cost of a false positive and false negative are low. In our deep-space mission context, a false positive would require the crew’s medical officer to simply review the ECG once the rhythm would be falsely flagged, and a false negative would not be very costly in a constant biomonitoring context with more occasions to flag arrhythmic patterns in subsequent 30-second windows.

As for operational performance, the ability to process the entire pipeline from raw data to inference in 9.2 s, thus less time than it takes for the sliding window to complete a 30-second recording, also indicated the efficiency of such an approach for an efficient real-time cardiac arrhythmia flagging system. The performance was not noticeably hindered by the presence of large amounts of noise thanks to variable mode deconstruction and reconstruction conserving the valuable signal information.

Table 3 shows the test set performance being systemically better than the training set, scoring higher in class-wise F1 scoring (cf. Table 3), since the model was then fully trained. It is not the case for the aggregated mean score of the cross validation since the first folds have low performance, lowering the average. However, this behavior does not translate to the weighted confusion matrix in Fig. 5 since this increase in performance, while notable, is overshadowed by the much smaller sample space.

As for feature importance, it puts emphasis on P-wave amplitude and HRV values since the absence of an organized P wave in atrial fibrillation or the presence of an atrial flutter wave in atrial flutter represents atrial activity which is a crucial component to AFIB and AFL^58^. Similarly, arrhythmias in general are characterized by fast heart rate, which is tracked in this model through the PRR20 and heart rate features.

**Fig. 6:**
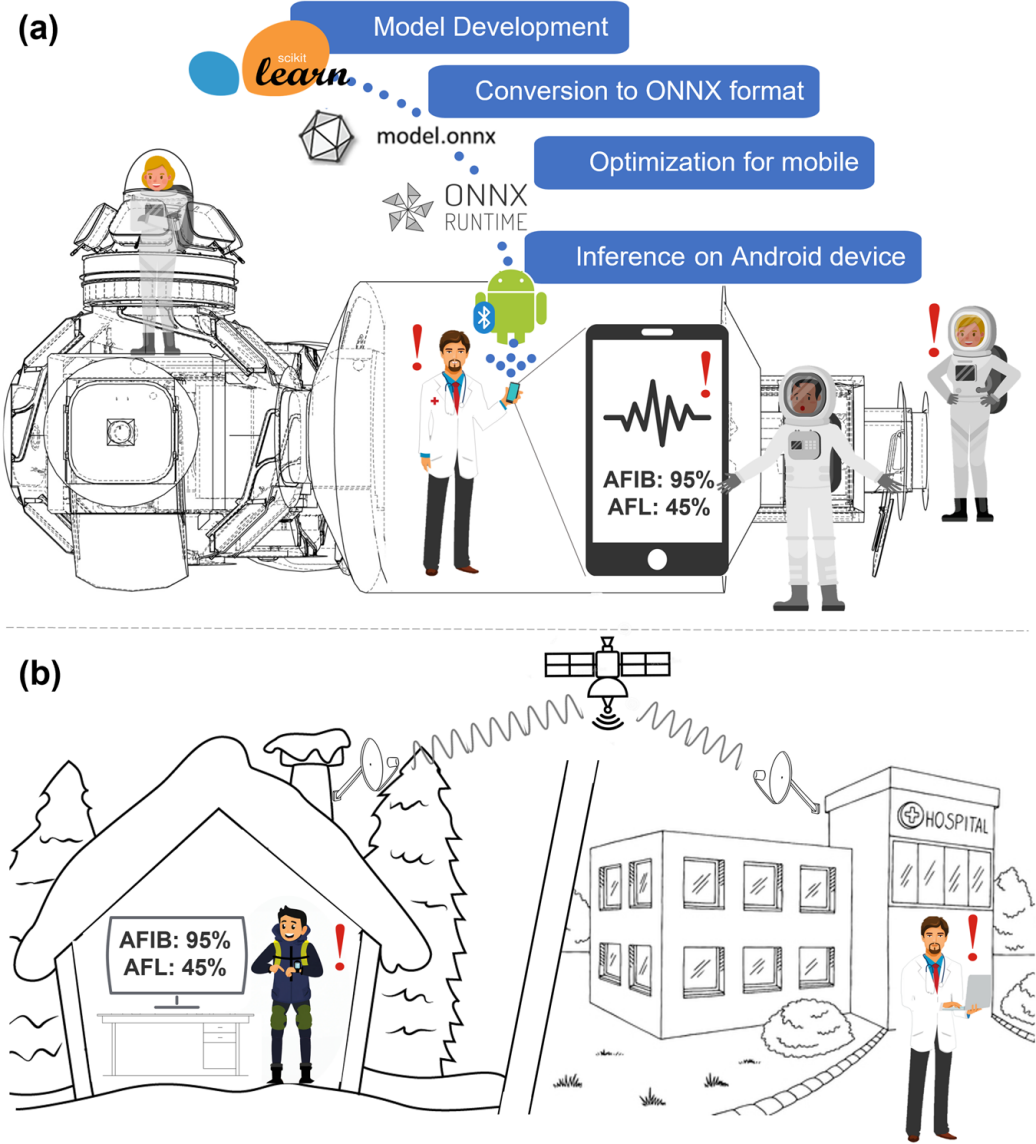
Model deployment examples for real-time health monitoring in different operational environments. This figure illustrates model deployment examples. **a** Model development and edge deployment process for deep-space operational context and CMO dashboard. The workflow includes model development with scikit-learn, conversion to ONNX format, optimization for mobile using ONNX Runtime, and inference on an Android device. This enables real-time monitoring of AFIB and AFL on a smartphone used by a medical officer aboard a spacecraft. **b** Similar deployment for remote biomonitoring of patients in areas with limited healthcare access. Data from a remote patient is transmitted via satellite to a hospital, allowing doctors to review results and provide timely interventions. This figure contains modified Shutterstock royalty-free stock images from a paid plan allowing for OA academic publication use under the Shutterstock Standard License, and modified iStockPhoto images from a free plan allowed for OA academic publication use under the iStockPhoto Standard License.

As shown in Table 4, the performance of this model is competitive and promising compared with relevant literature reporting similar use of ML workflows to identify a range of tachycardia on ECG datasets. While few papers study a combination of the same rhythms, combined group of datasets, training approach (nested cross-validation), evaluation metrics (AUROC, accuracy and F1 score), and edge computing inference—which illustrates the innovation in this work—the end inference performance can still be used as benchmark. Jekova et al.^59^ obtained a macro F1 score of 0.767 by using the PhysioNet 2021 Challenge database and a grid search over dense neural network (DenseNet) architectures over five training runs. Similarly to this work, their model gave more weight to the characteristics related to the P-wave pattern and heart rate values. On the same dataset, Plesinger et al.^60^ and Kamaleswaran et al.^61–65^ both used a Convolutional Neural Network approach but the latter with a Bagged Tree Ensemble approach, which respectively provided macro F1 scores of 0.70 and 0.81. Ojha et al.^30^ used a DL Long Short-Term Memory (LSTM) approach to obtain a macro F1 score of 0.927, slightly above the performance of our model. However, ML models typically require less computing power than DL models^66^, thus our approach stays competitive in our edge computing context where slightly better inference cannot be at a much higher computing cost. As an outlier in the literature that is mostly based on open-source datasets, Chang et al. used their own proprietary dataset of 65,932 samples of ECG to also train a long short-term memory model which gave a macro F1 score of 0.891. Their work could perhaps provide inspiration for a next stage of our research since their model tracked 12 arrhythmia pathologies thanks to the size of their proprietary dataset and the implicit control they have over it resulting in few rhythms with F1 scores under 0.9.^30,59,60,62–65^ Given that the macro F1 score of this work is above most values reported in the literature (cf. Table 4), we can also conclude that this overall approach which leverages methods in nested cross-validation loops, denoising and lightweight optimized edge inference through the ONNX format—a deployment process synthesized in Fig. 6a in the operational space environment—represents a gain in performance. This reinforces the decision to pursue with ML rather than DL to increase deployability and interpretability while maintaining high-performance inference. Our approach is also valuable specifically in medical research as our modular pipeline allows for easy integration of new pathologies and model architectures as it iterates on all features and updates the hyperparameters and parameters at once. This enables a specialized inference for each medical condition or pathology, which might depend on different features. There is no need to rebuild the pipeline for each new cardiovascular model based on ECG data. In fact, as mentioned in Section 2.5, the modular pipeline has demonstrated the capacity to train a different model architecture with only minor adjustments.

**Table 4:** Performance and characteristics of other ECG classification models in the literature

Pipeline	Rythms	Databases used	Macro F1 score	NSR F1 score	AFIB F1 score	AFL F1 score	Accuracy (%)
This study (Test set)	NSR, AFIB, AFL	See Databases Section	0.914	0.994	0.944	0.803	Mean: 99.1, NSR: 98.9, AFIB: 98.6, AFL: 99.7
Jekova et al. (2022)^59^	AFIB, AFL, others	PhysioNet/Cinc Challenge2021	0.767	--	--	--	93.8
Plesinger et al. (2017)^60^	NSR, AFIB, others	PhysioNet Challenge2017	0.81	0.91	0.8	--	--
Chang et al. (2021)^62^	NSR, AFIB, AFL, others	Proprietary 65,932 samples of 10 s	0.891	0.818	0.947	0.909	NSR: 96.5, AFIB: 99.1, AFL: 98.2
Petmezas et al. (2021)^63^	NSR, AFIB, AFL, others	AFDB	0.816	0.984	0.971	0.889	--
Kim et al. (2022)^64^	NSR, AFIB, AFL, others	1: MIDB 2: AFDB	Macro1: 0.916, Macro2: 0.932	0.997 0.995	0.829 0.958	0.983 0.992	99.2 99.35
Guan et al. (2021)^65^	NSR, AFIB, AFL, other	CUDB, MITDB, AFDB	0.962 0.927	0.994 0.973	0.989 0.993	0.925 0.926	99.2 99.3
Ojha et al. (2023)^30^	NSR, AFIB, AFL, VFIB	MITDB	0.927	0.915	0.991	0.974	99.2

A limitation of this ML pipeline stemmed from the scarcity of open-access ECG data obtained from space missions, as this data is often proprietary or confidential. Thus, this work depended on ECG data collected on Earth which may not perfectly replicate in-space conditions. However, using terrestrial ECG data from analogue noise and exercise contexts, including those from wearable devices, acted as a useful starting point for developing and testing a first deep-space ECG modeling tool and pipeline. Now proven with terrestrial data, this work, and its ML pipeline—unchanged by potential spaceflight ECG variations—can thus offer a replicable, interoperable, and innovative foundation for more accurate future model development trained on in-space ECG data when it becomes available.

Notwithstanding the model’s benefits outlined in section 4.2, it is also important to note that currently the development of the model—contrary to the edge inference—is extremely resource-intensive in time and computational power as the grid search and nested cross-validation approach is exhaustive in terms of solution space but also calculations. Thus, there is room for improvement in optimizing the development side of the model. A path to decrease this computational load might be in investigating less resource-intensive denoising techniques for low-noise situations where currently complex methods are used to clear small amounts of noise. A two-step approach could be beneficial. Rewriting the pipeline in a programming language closer to machine code (i.e., C, C++) could also be an improvement. The current pipeline also requires large memory bandwidth, which might not be possible on slower hard drives in legacy space hardware such as the ISS. With lightweight inference, the pipeline should follow suit.

As can be noticed in the class-wise F1 scoring, AFL was the weakest identified rhythm. This is for a variety of reasons. The main being that AFL is rare in the population with estimations of only 0.088% experiencing it per year^67^. ECG recordings containing it are thus very scarce, causing imbalanced datasets such as those used in this research. Additionally, AFL, when recorded is combined under AFIB^68^ or with AFIB and AFL under AF such as in Jekova et al.^59^ which studied binary classification AF/non-AF^59^. Additionally, the number of samples in Table 1 are generally lower than in literature on similar databases^69^, since it reflects the strict label conflict resolution method implemented to maximize classification performance, which is discussed in the pre-processing section. Another reason behind the weaker AFL scoring is that it is often misclassified as AFIB since they are quite similar. It is also widely described in literature that AFL generally has more inaccurate detection^59^. Due to this imbalance, accuracy was calculated but not used in evaluating the mode as a viable confidence metric. With unbalanced datasets, the model may achieve high accuracy by simply identifying the majority class all the time, while performing poorly on the minority class. This is because the contribution of each class to the accuracy is weighed equally, regardless of the actual number of examples in each class. F1 scores consider the relative importance of each class, making it a better metric for this study. A future study would benefit from more AFL recordings to balance the dataset.

While this solution addresses the emergent need for advanced point-of-care health systems in deep space, it is also very relevant for Canadian northern and remote communities. They experience similar situations of physical isolation, with sometimes very large distances to health centers, which make them unpractical and functionally impossible to reach on a regular basis for periodic screenings and for emergencies^70^. These communities also experience information isolation like astronauts on the far side of the moon as extreme weather events can incapacitate an already unreliable connectivity infrastructure for extended periods of time^71^. Thus, their experience of a flawed universal healthcare coverage could benefit from affordable at-home diagnostic tools. These can provide regular diagnostics to a central hospital center, which can then convene the patient when the situation requires their transfer such as depicted in Fig. 6b. This perspective is even more relevant in the context of the United States’ Food and Drugs Administration (FDA) approving of algorithms for human healthcare. A total of 58 out of 520 of FDA-approved algorithms are related to cardiology^72^. As for mass adoption in Earth devices and integration in space medical decision-support system, this algorithm would have to integrate interoperability standards.

Overall, this work demonstrates the performance of a decision tree classifier based on morphological and hear rate data and how its deployment and inference capability on edge android devices can facilitate the work of CMOs in extreme environments such as in deep space and in remote communities. The modular and easily upgradeable pipeline can be extended in future work to integrate different types of tachycardia and biometric sources to serve as a more holistic model of human health factors in space. Such a final model could be implemented in terrestrial remote healthcare contexts to stream wearable data, enable constant screening without having to go to a health center, facilitates physician online monitoring in addition to live telemedicine, and can justify in-person intervention when a suite of patterns is detected.

## Data Availability

The datasets analysed in this study are open source and published online at the URLs provided in the methods section along with each dataset description. No additional data collection was conducted on human subjects, thus complying with the Declaration of Helsinki.

## Code availability

The code used in the course of this study is available from the corresponding author upon reasonable request.

## References

[1] Smitherman, D. & Schnell, A. Gateway Lunar Habitat Modules as the Basis for a Modular Mars Transit Habitat. in *Aeroconf 2020*. 1-12 (IEEE, 2020).

[2] Doarn CR, Polk JD, Shepanek M (2019). Health challenges including behavioral problems in long-duration spaceflight. Neurol. India.

[3] Tran KA (2021). Evidence Supporting the Management of Medical Conditions During Long-Duration Spaceflight: Protocol for a Scoping Review. Jmir Res Protoc..

[4] Cheshier, L. *Artemis I – Flight Day Five: Orion Enters Lunar Sphere of Influence Ahead of Lunar Flyby*, https://blogs.nasa.gov/artemis/2022/11/20/artemis-i-flight-day-five-orion-enters-lunar-sphere-of-influence-ahead-of-lunar-flyby/ (2022).

[5] McBrayer Katherine, T. Communication Delays, Disruptions, and Blackouts for Crewed Mars Missions. in *ASCEND*. (ARC, 2022).

[6] Wooster, P., Braun, R., Ahn, J. & Putnam, Z. Trajectory Options for Human Mars Missions. in *AIAA/AAS ASCE*. 1-17 (AIAA, 2012).

[7] Krittanawong, C. et al. Human Health during Space Travel: State-of-the-Art Review. *Cells* **12**, 10.3390/cells12010040 (2022). 10.3390/cells12010040 PMC9818606 36611835

[8] Shibata S (2023). Cardiac Effects of Long-Duration Space Flight. J. Am. Coll. Cardiol..

[9] Shen M, Frishman WH (2019). Effects of Spaceflight on Cardiovascular Physiology and Health. Cardiol. Rev..

[10] Khine, H. W. et al. Effects of prolonged spaceflight on atrial size, atrial electrophysiology, and risk of atrial fibrillation. *Circ Arrhythm Electrophysiol* **11**, 10.1161/CIRCEP.117.005959 (2018). 10.1161/CIRCEP.117.005959 29752376

[11] Recommendations for Exploration Spacecraft Internal Atmospheres: The Final Report of the NASA Exploration Atmospheres Working Group. (NASA Johnson Space Center, National Aeronautics and Space Administration, 2010).

[12] Ercan E (2021). Effects of aerospace environments on the cardiovascular system. Anatol. J. Cardiol..

[13] Sasi SP (2017). Different sequences of fractionated low-dose proton and single iron-radiation-induced divergent biological responses in the heart. Rad. Res..

[14] Giacinto, O. et al. Cosmic Radiations and the Cardiovascular System: A Narrative Review. *Cardiol Rev*., 10.1097/CRD.0000000000000521 (2022). 10.1097/CRD.0000000000000521 36728769

[15] Anzai T, Frey MA, Nogami A (2014). Cardiac arrhythmias during long‐duration spaceflights. J. Arrhythm..

[16] Lee, S., Stenger, M. B., Laurie, S. S. & Macias, B. R. Risk of cardiac rhythm problems during spaceflight. Report No. JSC-CN-39745, (NASA, 2017).

[17] Barratt, M. R. & Pool, S. L. in *Principles of Clinical Medicine for Space Flight* 141-142 (Springer Science & Business Media, 2008).

[18] Verdino RJ (2015). Untreated atrial fibrillation in the United States of America: Understanding the barriers and treatment options. J. Saudi Heart Assoc..

[19] Krihak, M., Russell, B., Shetye, S. & Shaw, T. Exploration Medical Capability Clinical Decision Support System Concept of Operations. Report No. HRP-48033, (NASA, 2021).

[20] Jirak P (2022). How spaceflight challenges human cardiovascular health. Eur. J. Prev. Cardiol..

[21] Marois A, Salvan L, Lemaire N, Gagnon J-F (2023). User-Centred Dashboard for Sensors-Enabled Human State Monitoring: Two Operational Use Cases. Usabil. Use. Exp..

[22] Peberdy MA (2007). Recommended Guidelines for Monitoring, Reporting, and Conducting Research on Medical Emergency Team, Outreach, and Rapid Response Systems: An Utstein-Style Scientific Statement. Circulation.

[23] Romero E, Francisco D (2020). The NASA human system risk mitigation process for space exploration. Acta Astronaut.

[24] Garcia-Gomez, J. *Basic principles and concept design of a real-time clinical decision support system for autonomous medical care on missions to Mars based on adaptive deep learning*. (2020).

[25] Easter, B. *SysML Activity Diagram Emergency Care by a Non-CMO*, https://www3.nasa.gov/specials/hrp/exmc/clinical-decision-support-system-model/index.html?refid=a543b400-334b-460b-8fd7-ed3043d9aaa8 (2023).

[26] Easter, B. *Content Diagram Emergency Care by a Non-CMO*, https://www3.nasa.gov/specials/hrp/exmc/clinical-decision-support-system-model/index.html?refid=09a0421f-4e19-47a1-bbf9-9588d2d7e054 (2023).

[27] Hua H (2023). Edge Computing with Artificial Intelligence: A Machine Learning Perspective. Acm Comput Surv..

[28] Murshed MGS (2021). Machine Learning at the Network Edge: A Survey. Acm Comput Surv..

[29] Easter, B. *Requirement Table SE Level 4.1 w/ Relationships Table*, https://www3.nasa.gov/specials/hrp/exmc/clinical-decision-support-system-model/index.html?refid=4ef181d6-813b-4135-b500-03e7cf9e729a (2023).

[30] Ojha, M. K., Wadhwani, S., Wadhwani, A. K. & Shukla, A. Automated Identification of Tachyarrhythmia from Different Datasets of Heart Rate Variability Using a Hybrid Deep Learning Model. in *ICCI*. 159-167 (Springer Nature, 2023).

[31] Gaur M, Faldu K, Sheth A (2021). Semantics of the Black-Box: Can Knowledge Graphs Help Make Deep Learning Systems More Interpretable and Explainable?. IEEE Internet Comput..

[32] Aronow WS (2008). Treatment of atrial fibrillation and atrial flutter: Part II. Cardiol. Rev..

[33] Nesheiwat, Z., Goyal, A. & Jagtap, M. *Atrial Fibrillation*, https://www.ncbi.nlm.nih.gov/books/NBK526072/ (2023). 30252328

[34] Stiell IG (2021). 2021 CAEP acute atrial fibrillation/flutter best practices checklist. Can. J. Emerg. Med.

[35] Pruthi, S. *Atrial Flutter*, https://www.mayoclinic.org/diseases-conditions/atrial-flutter/symptoms-causes/syc-20352586 (2023).

[36] Md-Billal, H., Syed, K., Bashar, J. & Lazaro, N. A robust ECG denoising technique using variable frequency complex demodulation. *Comput. Methods Programs Biomed*. **200**, 10.1016/j.cmpb.2020.105856. (2020). 10.1016/j.cmpb.2020.105856 PMC7920915 33309076

[37] Moody G, Muldrow W, Mark R (1984). A noise stress test for arrhythmia detectors. CinC.

[38] ANSI/AAMI EC57: 2012—Testing and Reporting Performance Results of Cardiac Rhythm and ST Segment Measurement Algorithms. (Association for the Advancement of Medical Instrumentation (AAMI), American National Standard, 2013).

[39] Habib A (2019). Impact of ECG Dataset Diversity on Generalization of CNN Model for Detecting QRS Complex. IEEE Access.

[40] Moody G, Mark R (2001). The impact of the MIT-BIH Arrhythmia Database. IEEE Open J. Eng. Med. Biol..

[41] Moody G, Mark R (1983). A new method for detecting atrial fibrillation using R-R intervals. CinC.

[42] Petrutiu S, Sahakian A, Swiryn S (2007). Abrupt changes in fibrillatory wave characteristics at the termination of paroxysmal atrial fibrillation in humans. Europace.

[43] Liu FF, Liu CY, Zhao LN (2018). An open access database for evaluating the algorithms of ECG rhythm and morphology abnormal detection. J. Med. Imaging Health Info..

[44] Drew BJ (2004). Practice standards for electrocardiographic monitoring in hospital settings: an American Heart Association scientific statement from the Councils on Cardiovascular Nursing, Clinical Cardiology, and Cardiovascular Disease in the Young. Circulation.

[45] Robinson E, Clark D (1991). Sampling and the Nyquist frequency. Lead. Edge.

[46] Zhechev, Y., Kosteletskiiand, V. P. & Zabolotsky, A. M. in *Journal of Physics: Conference Series* (IOP Publishing, 2021).

[47] Lunar L1 Gateway Conceptual Design Report. (Advanced Development Office Advanced Design Team, National Aeronautics and Space Administration Lyndon B. Johnson Space Center, 2001).

[48] Malhotra V, Sandhu MK (2021). Electrocardiogram Signals Denoising Using Improved Variational Mode Decomposition. J. Med Signals Sens.

[49] Kaur, C., Bisht, A. & Singh, P. EEG Signal denoising using hybrid approach of Variational Mode Decomposition and wavelets for depression. *Biomed. Signal Process. Control* **65**, 10.1016/j.bspc.2020.102337 (2021).

[50] Stracina, T. & et al. Golden Standard or Obsolete Method? Review of ECG Applications in Clinical and Experimental Context. *Front in Phys*. **13**, 10.3389/fphys.2022.867033 (2022). 10.3389/fphys.2022.867033 PMC9082936 35547589

[51] Xu Q (2023). Interpretability of Clinical Decision Support Systems Based on Artificial Intelligence from Technological and Medical Perspective: A Systematic Review. J. Healthc. Eng..

[52] Schmitt, M. *Interpretable Machine Learning*, https://www.datarevenue.com/en-blog/interpretable-machine-learning (2021).

[53] Gupta, A., Banerjee, A., Babaria, D., Lotlikar, K. & Raut, H. in *ICSADL 2021* 527-538 (Springer Singapore, Thailand, 2021).

[54] Dora L, Agrawal S, Panda R, Abraham A (2018). Nested cross-validation based adaptive sparse representation algorithm and its application to pathological brain classification. Expert Syst. Appl.

[55] Swalin, A. *Choosing the Right Metric for Evaluating Machine Learning Models* https://medium.com/usf-msds/choosing-the-right-metric-for-evaluating-machine-learning-models-part-2-86d5649a5428 (2018).

[56] Lin, W.-F. et al. ONNC: A Compilation Framework Connecting ONNX to Proprietary Deep Learning Accelerators. in *AICAS 2019*. (IEEE, 2019).

[57] Streiner DL (2017). Statistics Commentary Series: Commentary No. 22: Setting Cut-Points: Receiver Operating Characteristics Analysis. J. Clin. Psychopharmacol..

[58] Portet, F. P wave detector with PP rhythm tracking: evaluation in different arrhythmia contexts. *Physiol. Meas*. **29**, 10.1088/0967-3334/29 (2008). 10.1088/0967-3334/29/1/010 18175865

[59] Jekova, I., Christov, I. & Krasteva, V. Atrioventricular Synchronization for Detection of Atrial Fibrillation and Flutter in One to Twelve ECG Leads Using a Dense Neural Network Classifier. *Sensors (Basel)* **22**, 10.3390/s22166071 (2022). 10.3390/s22166071 PMC9413391 36015834

[60] Plesinger, F., Nejedly, P., Viscor, I., Halamek, J. & Jurak, P. Automatic detection of atrial fibrillation and other arrhythmias in holter ECG recordings using rhythm features and neural networks. in *2017**CinC*. 1-4 (IEEE, 2018).

[61] Kamaleswaran, R., Mahajan, R. & Akbilgic, O. A robust deep convolutional neural network for the classification of abnormal cardiac rhythm using single lead electrocardiograms of variable length. *Physiol. Meas*. **39**, 10.1088/1361-6579/aaaa9d (2018). 10.1088/1361-6579/aaaa9d 29369044

[62] Chang K-C (2021). Usefulness of machine learning-based detection and classification of cardiac arrhythmias with 12-lead electrocardiograms. CJC.

[63] Petmezas G (2021). Automated atrial fibrillation detection using a hybrid CNN-LSTM network on imbalanced ECG datasets. Biomed. Signal Process. Control.

[64] Kim YK, Lee M, Song HS, Lee S-W (2022). Automatic Cardiac Arrhythmia Classification Using Residual Network Combined With Long Short-Term Memory. IEEE Trans. Instrum. Meas..

[65] Guan Y, Xu J, Liu N, Wang J, An Y (2021). ECG Arrhythmia Detection Based on Hidden Attention Residual Neural Network. Bioinf. Res. Appl..

[66] KavlakogluE. A. I. *vs*. *Machine Learning vs. Deep Learning vs*. *Neural Networks: What’s the Difference?*, https://www.ibm.com/cloud/blog/ai-vs-machine-learning-vs-deep-learning-vs-neural-networks (2020).

[67] Granada J (2000). Incidence and predictors of atrial flutter in the general population. J. Am. Coll. Cardiol..

[68] Zhang H (2023). MaeFE: Masked Autoencoders Family of Electrocardiogram for Self-Supervised Pretraining and Transfer Learning. IEEE Trans. Instrum. Meas..

[69] Wang, J. Automated detection of atrial fibrillation and atrial flutter in ECG signals based on convolutional and improved Elman neural network. *KBS* **193**, 10.1016/j.knosys.2019.105446 (2020).

[70] Kent, A. E. *Closing the northern gap: care provider perspectives on the suitability of an eHealth app for maternal mental health in Northwestern Ontario* Master of Health Sciences thesis, Lakehead University, (2020).

[71] Intahchomphoo C (2018). Indigenous peoples, social media, and the digital divide: A systematic literature review. AICRJ.

[72] Fornell, D. *FDA has now cleared more than 500 healthcare AI algorithms*, https://healthexec.com/topics/artificial-intelligence/fda-has-now-cleared-more-500-healthcare-ai-algorithms (2023).

